# Influence of caries and molar incisor hypomineralization on oral health-related quality of life in children

**DOI:** 10.1007/s00784-021-03828-5

**Published:** 2021-07-14

**Authors:** Lucas Michaelis, Markus Ebel, Katrin Bekes, Christian Klode, Christian Hirsch

**Affiliations:** 1 Paediatric Dentistry Practice Leo Löwenzahn, Department of Paediatric Dentistry, University of Leipzig, Liebigstr.12, 04103 Leipzig, Germany; 2 Department of Paediatric Dentistry, University Clinic of Dentistry, Medical University Vienna, Sensengasse 2a, 1090 Vienna, Austria; 3 Department of Business Analytics and Data Science, HMKW University of Applied Science, Höninger Weg 139, 50969 Köln, Germany; 4 Faculty of Economics and Management, Department of Knowledge Management, University of Marburg, Universitätsstraße 25, 35037 Marburg, Germany; 5 Department of Paediatric Dentistry, School of Dentistry, University of Leipzig, Liebigstr. 12, 04103 Leipzig, Germany

**Keywords:** Caries, MIH, Child Perceptions Questionnaire, CPQ8-10, OHRQoL

## Abstract

**Objectives:**

This study was aimed to compare the impact of caries and molar incisor hypomineralization (MIH) on oral health-related quality of life (OHRQoL) in children.

**Material and methods:**

A total of 528 German children aged 7 to 10 years were recruited, half affected by caries and the other half affected by MIH. Both groups were matched according to age, sex, and social status and divided into 3 categories according to severity. The German version of the Child Perceptions Questionnaire for 8- to 10 years old (CPQ-G8-10) was used to analyze the impact on OHRQoL by applying ANOVA models.

**Results:**

Patients with MIH showed a mean CPQ score of 10.7 (± 9.3). This was significantly higher compared to the caries group with 8.1 (± 9.8). The score increased linearly from the low severity category to the high severity category in both groups (caries, 4.1 to 13.8; MIH, 5.2 to 17.7, respectively).

**Conclusion:**

With increasing severity, both clinical conditions showed a greater negative impact on OHRQoL. MIH was associated with more impairments.

**Clinical relevance:**

Currently, the focus in pediatric dentistry is placed on the prevention and treatment of caries. Both diseases may have a negative influence on OHRQoL. Since children perceive the impairments by MIH as worse and the prevalence is equal to that of caries, which focus might be shifted in the future.

## Introduction

Over the past few decades, there has been a major decrease in the prevalence of caries in children and teenagers in Western industrial nations. From 1989 to 2014, the average “decayed, missing, and filled teeth” index of 12 years old in Germany dropped from 4.1 to 0.5 [1]. Only 12.4% of those children had caries-free dentition in 1989 [2]. Due to intensive individual and group prophylaxis, the morbidity of this disease has been reduced substantially. Currently, 81.3% of 12 years old in Germany are caries-free [1]. However, this disease remains a worldwide problem and a public health challenge, and social inequalities in oral conditions continue to exist [3, 4].

Hypomineralized teeth, which clinically resemble teeth affected by molar incisor hypomineralization (MIH), were first described in 1987 [5]. During the 2003 European Academy of Pediatric Dentistry (EAPD) meeting, “molar incisor hypomineralization” was proposed as its own clinical entity for the first time, replacing the term “idiopathic enamel hypomineralization” [6, 7]. According to the 2016 Fifth German Oral Health Study, the prevalence of MIH among 12 years old peaked at around 30%, which was higher than the prevalence of caries seen in children of the same age group [1]. Regardless of which disease is more common, both can have a major influence on children’s oral health-related quality of life (OHRQoL) [8–10]. If either reaches a certain severity, both caries and MIH can cause symptoms such as hypersensitivity or pain, leading to impaired chewing and food intake [11–13]. The resulting psychosocial burden is also a side effect that should not be neglected. Chronic pain can cause difficulties in concentration and, therefore, could negatively impact children’s capabilities in school [14]. In addition, both diseases can cause esthetic issues, which might influence the emotional state of the child. In the past, research has mainly focused on collecting objective diagnostic parameters. Recently, researchers have paid more attention to subjective evaluations, for which standardized questionnaires have proven to be very helpful. For children, the Child Perceptions Questionnaire (CPQ8-10, CPQ11-14) is frequently used [15, 16].

To date, only a few studies have investigated the influence of both caries and MIH on the oral health of children in this age group. In 2016, Mota-Velosa et al. [12] analyzed how untreated caries impacted the quality of life of children. The participants showed a significant decrease in OHRQoL as tooth decay increased [13]. Other authors have focused on the effect of MIH on the quality of life of children in this age group. Both Velandia et al. [17] and Gutiérrez et al. [18] showed that MIH significantly deteriorates the OHRQoL of the probands. However, to date, no research has examined and compared the influence of caries and MIH on children’s OHRQoL matched by the degree of severity. This study was aimed to determine which condition has a greater impact on the OHRQoL of affected children.

## Materials and methods

### Key elements of study design

In this matched pairs study, the impact of caries and MIH on OHRQoL in 7- to 10-year-old children was investigated. To evaluate the influence of both diseases, their clinical severity was additionally considered. By categorizing a consecutive sample of children according to various criteria, it was possible to compare the clinical conditions.

### Setting

Patients were recruited from a pediatric dental clinic in Bergisch Gladbach, North Rhine-Westphalia, Germany, between January and November 2019. All employees of the dental clinic, which included dentists, dental hygienists, and dental assistants, were briefed about the details of the study and asked to recruit patients. One leading calibrated examiner (LM) performed the clinical examinations. The children were diagnosed based on clinical and radiological examinations and their teeth were evaluated under artificial light using an air/water syringe, a dental mirror, and a standardized probe.

### Participants

Children aged between 7 and 10 years who had at least one tooth affected by caries or MIH were included. However, patients who presented with both diseases were excluded.

For the initial detection of caries, laser-induced-fluorescence with a Kavo-DIAGNOdent pen was used, together with visual-tactile examination. Once a tooth was possibly affected by caries, a radiograph was taken and the presence and extent of the lesion was evaluated using the judgment criteria provided by the World Health Organization (WHO) and American Dental Association (ADA) [19, 20]. The WHO categorizes teeth as having either a sound tooth structure or a decayed crown. A crown is considered sound if there is no evidence of treated or untreated dental caries. Teeth in a stage of caries that precedes cavitation and those with other conditions similar to the early stages of caries are considered sound, because they cannot reliably be clinically diagnosed. Caries is considered present when a lesion in a pit, fissure, or on a smooth tooth surface has an unmistakable cavity, undermined enamel, or a detectably softened floor or wall. If a tooth with caries is detected, it is further evaluated using the ADA criteria [19]. This classification ranks teeth from a sound tooth structure to a lesion with advanced caries. Teeth are considered sound if they show normal translucency and glossiness and do not have a clinically detectable lesion or if they have an intact restoration/sealant. Radiographically, no translucency can be observed. A tooth is considered to have an initial carious lesion if it is limited to the enamel/cementum or the outermost layer of dentin. Clinically, a white-to-brown change in color is seen, but there is no dark shadow indicating major dentin involvement. Radiographically, an initial lesion must only extend to the dentin-enamel junction or the outer one-third of the dentin. A moderate lesion is detected if the enamel is visibly lost or if shadowing or translucency is present and the surface shows some cavitation. Advanced lesions have a full enamel cavitation, clinically exposing the dentin. Radiographically, these lesions extend to the inner third of the dentin, reaching the circumpulpal area.

To establish a calibrated diagnosis for teeth with MIH, the authors used the EAPD criteria [6, 7], which requires the teeth to have a clearly definable white/yellow-to-brown discolored opacity of at least 1 mm in diameter on the smooth surface. With increasing severity, there is a higher tendency for post-eruptive hypersensitivity, especially in the affected molars. Teeth with atypical restorations and permanent teeth that were inexplicably extracted were diagnosed with MIH. Subsequently, all hypomineralized teeth were divided into 3 categories according to severity based on the classification developed by Mathu-Muju and Wright in 2006 [21]. In order to determine the OHRQoL, children were only included in this study if they could complete the CPQ-G8-10 without their caregiver’s assistance. Moreover, all children who had any type of illness in the previous 4 weeks that could influence the oral findings, such as sinusitis or otitis media, were excluded. Children who received orthodontic treatment and those with orthodontic anomalies, such as crowding, crossbite, open bite, or any type of malocclusion in general, were also excluded, since these could influence their OHRQoL. Children with other dental anomalies, including bruxism, secondary caries, enamel hypoplasia, and dental or gingival trauma, were also excluded. The study was approved by the ethics committee of the University of Leipzig (AZ: 152/19-ek).

### Sample Size

A total of 528 children (266 boys and 262 girls) were included in this matched pairs study. A G*power analysis (www.gpower.hhu.de) resulted in a total of 88 patients for each of the three severity categories, meaning 264 children in the caries and the MIH group, equaling a total of 528 patients.

### Variables

The following variables were investigated: age (in years), sex (male/female), and social status according to Winkler and Stolzenberg (low, middle, and high) [22]. The caries were divided into initial, moderate, and advanced lesions according to the ADA classification [19]. EAPD criteria were used to define the severity of MIH, divided into mild, moderate, and severe [6]. The first quantitative outcome variable was the OHRQoL, which was measured using the German version of the CPQ8-10 [23]. The CPQ-G8-10 contains 25 questions focusing on the respondent’s oral health and well-being over the prior 4 weeks. Each question is rated on a scale from 0 to 4, with 0 indicating no impact on OHRQoL and 4 indicating the most negative impact. The control variables were gender, social status, and age group. The caries/MIH groups served as predictors.

### Data sources and measurements

The dental assistants instructed all the children on the format of the study. After obtaining official consent from the children’s legal guardians, the questionnaires were distributed, followed by clinical investigation. When a patient proved to be a possible candidate for this study, the child and caregiver were asked about any prior illnesses in the previous 4 weeks that could possibly influence OHRQoL. If any were detected, the patient was excluded from the study. Finally, the questionnaire was examined for completeness, and if answers were missing or incorrect, the child was consulted for correction. If the clinical investigation and anamnestic data demonstrated that the child was a possible participant, LM digitally collected the data concerning the aforementioned variables. To ensure the patient’s anonymity, every participant’s name was replaced with a randomly assigned number.

Due to the varying clinical severity of caries and MIH, patients were divided into different categories according to severity. First, the participants were identified as either caries or MIH probands. To divide the MIH patients, a recently developed scheme was applied [12]. For the caries patients, a modified classification based on the ADA judgment criteria was used [18] and the scheme by Ebel et al [12]. The aim was to assign risk points to any individual tooth that reflected the clinically detected severity. By adding all the risk points, the calculated individual severity score placed the children in the appropriate severity category. Patients with a severity score of 0 were included in the low-severity category (LSC), patients with a severity score of 2–4 in the medium-severity category (MSC), and patients with a severity score > 4 in the high-severity category (HSC), depending on the clinical findings. Unaffected or mildly-affected MIH teeth were assigned 0 risk points because they do not cause hypersensitivity [12]. The same accounted for teeth affected by caries only located in the enamel. Moderately affected MIH teeth as well as teeth that showed caries in the outer to the middle third of the dentin were assigned 2 risk points. Severely affected MIH teeth and teeth with profound caries that had reached the inner third of the dentin were assigned 3 risk points. The children were then matched according to age, gender, and social status.

### Bias

The children’s emotional state could have been affected by the presence or absence of caregivers while completing the questionnaire. While the caregivers were asked not to help with the answers, the questionnaires were completed in the waiting room so this could not be controlled. Moreover, general diseases could also unknowingly have impacted the child’s emotional and social state and consequently could have increased the overall CPQ score.

### Statistical methods

The CPQ-G8-10 score was used to set up our analyses, and the subsequent dimensions (oral symptoms, functional limitations, emotional well-being, and social well-being) were used as additive indices within the analysis of variance (ANOVA) procedure as dependent quantitative variables. The authors conducted several analyses to test for significant mean differences between group A (patients with caries) and group B (patients with MIH) by using the matched pairs approach, where similar patients were compared (according to age, sex, and social status). These tests were controlled according to 3 severity groups (low, medium, and high). Then, 95% confidence intervals (CI) and *p*-values were computed and considered mean difference tests significant if the confidence intervals between the two groups did not overlap or if the *p*-values were less than 0.05. The same criteria for significance were also applied to all mean difference tests for the control variables of gender, age, and social status.

## Results

### Participants

In total, 528 children aged 7 to 10 years participated in the present study (Table 1). The mean age was 8.4 years (± 1.1 years), and 49% (*N* = 262) of the participants were female. Of all the children included in the study, 423 (80%) were Caucasian. Almost half (49%) of the participants were from the middle class, while 28% were from the lower social class, and 23% were from the higher social class, according to Winkler and Stolzenberg’s [22] classification.

**Table 1:** Summary of Sample Data (*n* = 528 patients)

	% (*n*)
Gender	
Male ♂	50.4 (266)
Female ♀	49.6 (262)
Social status	
Low	28.0 (148)
Medium	49.2 (260)
High	22.7 (120)
Ethnicity	
Caucasian	80.1 (423)
Non-Caucasian	19.9 (105)
Age	
Mean	8.4 years
Range	7–10 years
7 years	30.3 (160)
8 years	24.2 (128)
9 years	23.5 (124)
10 years	22.0 (116)

### Main results

A total of 12,217 teeth were included, 6,103 of which belonged to the caries group and 6,114 to the MIH group. In the caries group, 3,027 teeth were permanent and 3,076 were deciduous, whereas in the MIH group, 3,085 were permanent and 3,029 were deciduous teeth (Appendix Table 8).

We differentiated all caries- and MIH-affected teeth according to the type of dentition and the degree of clinical severity, as shown in Table 2. Of all examined teeth, 1,159 had carious lesions and 958 were affected by MIH. Most of the carious teeth were deciduous (*n* = 1,036; 89.4%), while only 123 (10.6%) were permanent. In contrast, most of the MIH-affected teeth were permanent (*n* = 827; 86.3%), and only 131 (13.7%) were deciduous.

**Table 2:** Summary of data on 2,117 teeth with caries (n = 1159) and MIH (*n* = 958) according to the American Dental Association Caries Classification by Young et al. [19] and the MIH Classification by Mathu-Muju and Wright [21]

2,117 teeth									
% (*n*)									
		MIH					Caries		
		45.3 (958)					54.7 (1,159)		
		I	II	III			I	II	III
**Deciduous teeth**	**13.7 (131)**	14.2 (88)	19.7 (41)	1.5 (2)	**Deciduous teeth**	**89.4 (1,036)**	83.7 (522)	94.2 (290)	98.7 (224)
**Permanent teeth**	**86.3 (827)**	85.8 (532)	80.3 (167)	98.5 (128)	**Permanent teeth**	**10.6 (123)**	16.3 (102)	5.8 (18)	1.3 (3)
**Total**	**100.0 (958)**	64.7 (620)	21.7 (208)	13.6 (130)	**Total**	**100.0 (1,159)**	53.8 (624)	26.6 (308)	19.6 (227)

*MIH* molar incisor hypomineralization

**Fig. 1:**
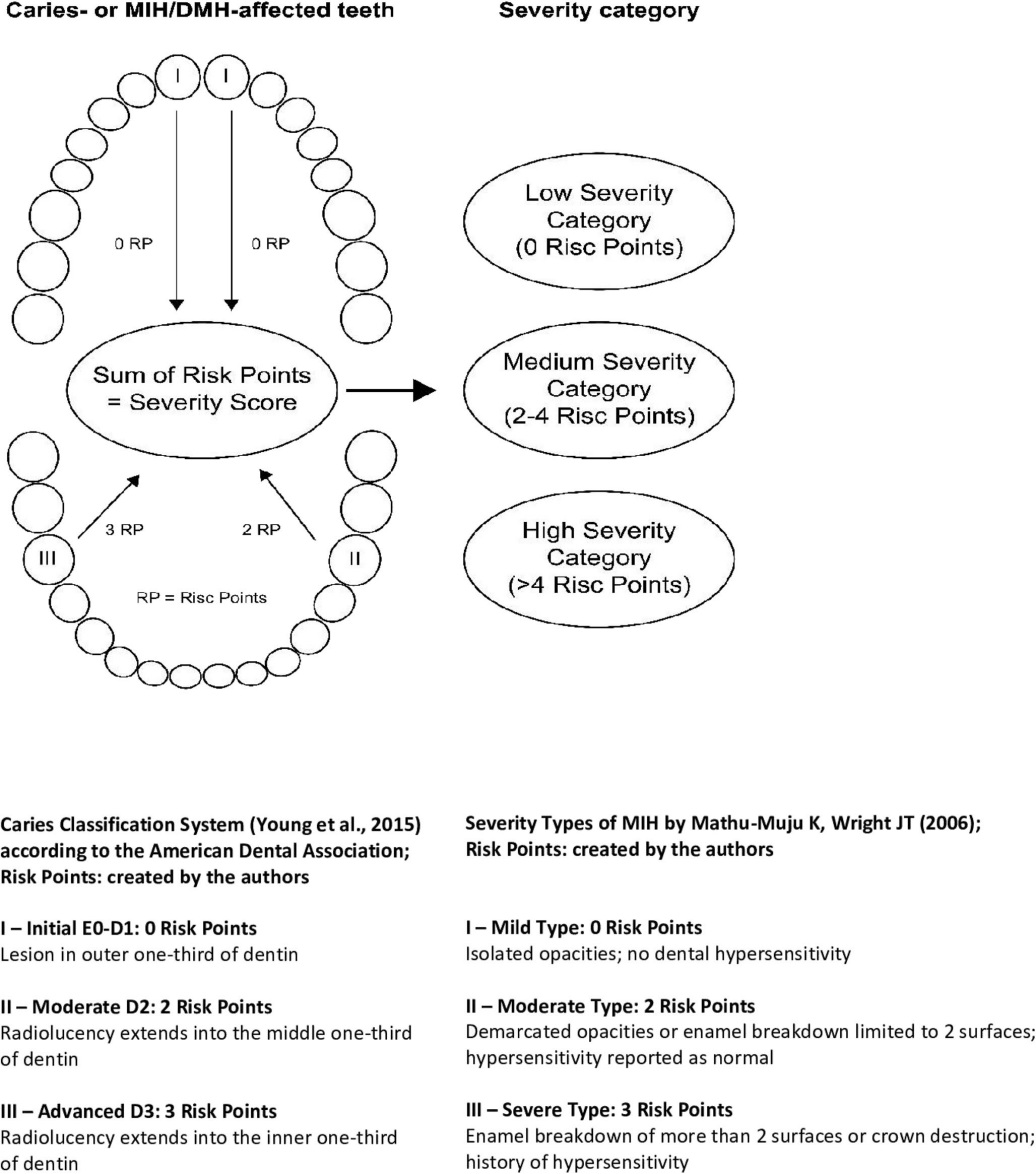
Schematic presentation of the severity score of teeth with caries or molar incisor hypomineralization (MIH)

According to the ADA’s [19] classification of caries-affected teeth, 624 teeth (53.8%) had initial carious lesions (Group I), 308 teeth (26.6%) had moderate lesions (Group II) that reached the middle third of the dentin, and 227 (19.6%) had advanced lesions (Group III), which reached the circumpulpal dentin (Fig. 1). Due to the selection criteria, none of the children in these groups had structurally compromised teeth in the form of MIH/deciduous molar hypomineralization (DMH).

Additionally, 958 teeth had a positive MIH diagnosis according to the EAPD criteria [6]. Based on the definition by Mathu-Muju and Wright [21], 20 of the teeth (64.7%) were mildly affected by MIH, 208 (21.7%) were moderately hypomineralized, and 130 (13.6%) teeth were severely affected. See Appendix Table 8 for additional information on teeth not captured by these classifications.

The relationship between the affected teeth according to dentition (primary and permanent) and position (frontal and posterior teeth) is presented in Table 3. In general, posterior teeth were more often affected than anterior teeth.

**Table 3:** Affected teeth in the MIH and caries groups

	All 2,117 affected teeth	
	MIH	Caries
	% (*n*)	
	45.3 (958)	54.7 (1,159)
Anterior teeth and canines, 18.8 (399)		
Deciduous 25.3 (101)	9.2 (26)	65.2 (75)
Permanent 74.7 (298)	90.8 (258)	34.8 (40)
Total	29.6 (284)	9.9 (115)
Posterior teeth (deciduous molars, premolars, molars), 81.2 (1,718)		
Deciduous 62.0 (1,066)	15.6 (105)	92.0 (961)
Permanent 38.0 (652)	84.4 (569)	8.0 (83)
Total	70.4 (674)	90.1 (1,044)

*MIH* molar incisor hypomineralization

In the caries group, the majority of the affected teeth were deciduous posterior teeth (*n* = 961; 92%), followed by permanent posterior teeth (*n* = 83; 8%). Similarly, more deciduous anterior teeth and canines were affected (*n* = 75; 65.2%) than permanent anterior teeth and canines (*n* = 40; 34.8%).

By far, the most affected teeth in the MIH group were permanent posterior (*n* = 569; 84.4%) and anterior teeth (*n* = 258; 29.6%). When deciduous teeth were affected by MIH, they were 4 times more likely to be posterior teeth (*n* = 105; 15.6%) than incisors or canines (*n* = 26; 9.2%).

An increase in the mean CPQ-G8-10 value was associated with an increase in the severity category of the children. Both diseases were portrayed individually, and the results were presented according to the respective CPQ8-10 subcategories (Table 4).

**Table 4:** Means and confidence intervals for CPQ Domain Scores according to caries and MIH severity categories

		LSC			MSC			HSC			Global DIFF
Group	CPQ Domain	Mean CPQ	95% CI		Mean CPQ	95% CI		Mean CPQ	95% CI		Test
			Lower	Upper		Lower	Upper		Lower	Upper	*p*-value
Caries	Oral symptoms	3.3	2.6	4.0	4.6	3.9	5.3	6.6	5.9	7.3	0.000
	Functional limitations	0.2	-0.3	0.7	0.8	0.3	1.3	2.9	2.4	3.4	0.000
	Emotional well-being	0.6	0.0	1.1	1.0	0.5	1.5	2.8	2.3	3.3	0.000
	Social well-being	0.0	0.0	0.1	0.0	0.0	0.1	1.4	0.7	2.1	0.011
	Total CPQ	4.1	2.2	6.0	6.3	4.5	8.2	13.8	11.9	15.7	0.000
MIH	Oral symptoms	1.4	0.9	2.0	3.6	3.1	4.1	6.4	5.9	7.0	0.000
	Functional limitations	0.1	-0.4	0.6	2.5	2.0	3.0	4.9	4.4	5.4	0.000
	Emotional well-being	2.9	2.1	3.6	2.7	2.0	3.4	5.0	4.3	5.8	0.000
	Social well-being	0.8	0.4	1.1	0.5	0.2	0.9	1.3	0.9	1.7	0.000
	Total CPQ	5.2	3.5	6.8	9.3	7.6	10.9	17.7	16.0	19.3	0.000

Considered significant if *p* < 0.05. *LSC* low-severity category, *MSC* medium-severity category, *HSC* high-severity category, *CPQ* Child Perceptions Questionnaire, *MIH* molar incisor hypomineralization, *CI* confidence interval

Regarding the total CPQ and all subcategories, the values significantly increased according to the severity categories for both diseases. In the caries group, the total CPQ-G8-10 scores increased from 4.1 in the LSC to 6.3 in the MSC and 13.8 in the HSC groups. When comparing the CPQ-G8-10 in all subcategories, the disease was shown to mostly manifest as oral symptoms, since these had the highest values. As with the total CPQ values, there was a constant increase in accordance with the disease severity (from 3.3 in the LSC to 6.6 in the HSC). Children in the HSC had toothaches twice as often as children in the LSC.

For the MIH group, the total CPQ-G8-10 values increased from 5.2 in the LSC to 9.3 in the MSC and 17.7 in the HSC. Similarly, for the CPQ subcategories, the scores tended to increase with each severity category. The most obvious increase was in the manifestation of oral symptoms (from 1.4 to 3.6 and 6.4), similar to the caries group. In the HSC, functional limitation scores also increased significantly from 0.1 to 2.5 and 4.9 (Table 4).

One very important aspect of this study was the statistical comparison between the MIH and caries groups in terms of the total CPQ and its subcategories (Table 5). Generally, the children in the caries group had lower values (with an average total CPQ value of 8.1) than the children in the MIH group (with an average total CPQ score of 10.7). Nonetheless, the oral symptoms category in the caries group was more strongly affected in all severity categories than that in the MIH group. However, the differences in the mean CPQ value in the subcategories were rated as minimal and not substantial.

**Table 5:** Means and confidence intervals for CPQ Subscale Scores according to caries and MIH severity categories

Severity Category	CPQ Subscale	MIH			Caries			Caries vs. MIH
		Mean CPQ	95% CI		Mean CPQ	95% CI		DIFF Test
			Lower bound	Upper bound		Lower Bound	Upper Bound	*p*-value
LSC (*n* = 176)	Oral symptoms	1.4	0.9	2.0	3.3	2.6	4.0	0.000
	Functional limitations	0.1	-0.4	0.6	0.2	-0.3	0.7	0.128
	Emotional well-being	2.9	2.1	3.6	0.6	0.0	1.1	0.000
	Social well-being	0.8	0.4	1.1	0.0	0.0	0.1	0.025
	Total CPQ	5.2	3.5	6.8	4.1	2.2	6.0	0.000
MSC (n = 176)	Oral symptoms	3.6	3.1	4.1	4.6	3.9	5.3	0.014
	Functional limitations	2.5	2.0	3.0	0.8	0.3	1.3	0.000
	Emotional well-being	2.7	2.0	3.4	1.0	0.5	1.5	0.000
	Social well-being	0.5	0.2	0.9	0.0	0.0	0.1	0.000
	Total CPQ	9.3	7.6	10.9	6.3	4.5	8.2	0.001
HSC (n = 176)	Oral symptoms	6.4	5.9	7.0	6.6	5.9	7.3	0.813
	Functional limitations	4.9	4.4	5.4	2.9	2.4	3.4	0.000
	Emotional well-being	5.0	4.3	5.8	2.8	2.3	3.3	0.001
	Social well-being	1.3	0.9	1.7	1.4	0.7	2.1	0.786
	Total CPQ	17.7	16.0	19.3	13.8	11.9	15.7	0.050
Total (n = 528)	Oral symptoms	3.8	3.4	4.2	4.8	4.4	5.3	0.000
	Functional limitations	2.2	2.1	2.9	1.3	1.0	1.6	0.000
	Emotional well-being	3.5	3.1	4.0	1.5	1.1	1.8	0.000
	Social well-being	0.9	0.7	1.1	0.5	0.3	0.7	0.025
	Total CPQ	10.7	9.6	11.8	8.1	6.9	9.3	0.000

Considered significant if *p* < 0.05. *LSC* low-severity category, *MSC* medium-severity category, *HSC* high-severity category, *CPQ* Child Perceptions Questionnaire, *MIH* molar incisor hypomineralization, *CI* confidence interval

Data analysis revealed no significant differences in the overall CPQ values depending on age and gender (Appendix Tables 9 and 10). However, significant differences were detected within the caries group in terms of social class. The mean CPQ value in the lower social class (14.1) was almost 4 times that of the higher social class and almost twice as high as the middle social class. In the MIH group, there were no significant differences in the mean value of the overall CPQ score. In this group, the mean value ranged from 10.9 in the high social class to 9.8 in the middle and 12.2 in the lower social classes (Appendix Table 11). In addition, the maximal total CPQ score was divided into 7 sub-scores, ranging from 0 to over 40, as shown in Table 6. These results demonstrated that 10% of the children in both groups had no OHRQoL issues and the CPQ-G8-10 rating was 0 points.

**Table 6:** Range of total CPQ scores (*n* = 528 patients)

CPQ scores	MIH	Caries
	*n* = 264	*n* = 264
	% (*n*)	
0	10.6 (28)	9.1 (24)
1-5	25.4 (67)	42.0 (111)
6-10	21.2 (56)	26.9 (71)
11-20	29.5 (78)	14.0 (37)
21-30	9.1 (24)	3.8 (10)
31-40	3.4 (9)	1.9 (5)
> 40	0.8 (2)	2.3 (6)
max	51	64

*CPQ* Child Perceptions Questionnaire, *MIH* molar incisor hypomineralization

In the caries group, most of the children (*n* = 206; 78%) had CPQ scores ≤ 10, with the majority in the range of 1–5 (*n* = 111; 42%). Only 58 children in this group (22%) had scores above 10. However, the overall highest CPQ score in the caries group was 64, while the highest in the MIH group was 51.

In general, the participants in the MIH group had higher total CPQ scores. The number of participants scoring > 10 was almost double that in the caries group (*n* = 113; 43% vs. *n* = 58; 22%, respectively). Additionally, in the MIH group, 57% (*n* = 151) scored ≤ 10, and most participants scored in the range of 1–20 (*n* = 78; 29%). Very few children scored over 40 (*n* = 2; 0.8%).

Furthermore, the ratings for the introductory questions of the CPQ8-10 concerning the participants’ overall well-being and oral health were examined (Table 7). Most of the children in the caries group rated their overall well-being as “very good” (51.1%; *n* = 135) or “good” (26.9%; *n* = 71). The questions on their oral health generated a similar response.

**Table 7:** Questions concerning overall well-being^†^ and oral health^‡^ (*n* = 528 patients)

	MIH (*n* = 264)		Caries (*n* = 264)	
	Overall well-being	Oral health	Overall well-being	Oral health
	% (*n*)		% (*n*)	
Excellent	10.2 (27)	1.5 (4)	11.7 (31)	3.8 (10)
Very good	77.7 (205)	28.8 (76)	51.1 (135)	32.2 (85)
Good	10.6 (28)	23.9 (63)	26.9 (71)	35.6 (94)
Moderate	1.5 (4)	41.3 (109)	9.5 (25)	20.8 (55)
Poor	0.0 (0)	4.5 (12)	0.8 (2)	7.6 (20)

^†^How would you rate your overall well-being?

^‡^How would you rate your oral health?

*MIH* molar incisor hypomineralization

Of the children with hypomineralized teeth, 87.9% (*n* = 232) described their overall well-being as “excellent” or “very good,” which was much higher than the same rating in the caries group (*n* = 166; 62.8%). No participant rated their overall well-being as “poor.”

Most children in the MIH group rated their oral health as “moderate” (*n* = 109; 41.3%), while 30.3% (*n* = 80) rated their oral health as “excellent” or “very good,” which was less than that in the caries group (*n* = 95; 36%).

## Discussion

Caries and MIH are the most common diseases of the orofacial system for children in the age group analyzed [1]. The CPQ-G8-10 by Bekes et al. [23], which is based on the questionnaire developed by the Canadian author Jokovic [16], was used to examine which of these conditions had a greater impact on the OHRQoL of children. The overall results showed that for both diseases, as clinical severity increased, subjectively perceived OHRQoL significantly worsened. Furthermore, the oral health of children in the MIH group was more affected than that of children with caries within the same severity category.

For both groups, the classification was based on the assumption that as more teeth were affected (per participant), and as the severity of each condition increased, the influence on the OHRQoL would be greater. Even though this assumption seems to be reasonable, currently, only two studies have analyzed OHRQoL using certain risk points for MIH patients [11, 12]. To date, no sufficient data are available for proband’s caries, which is why a scheme with certain risk points based on the ADA classification was developed for caries-affected teeth in this study. The existing data have only compared children with approximately equal clinical severity.

Moreover, in this study, additive effects were ruled out by ensuring that the children were either affected by caries or MIH but never both. Children with other diseases of the orofacial system were excluded so that only the impact of the investigated diseases on the OHRQoL was recorded. There were no significant differences in the answers and results of the CPQ-G8-10 according to gender or age.

However, the social status of the patients had a significant effect on the CPQ score in the caries group. Children classified as having a low social status according to Winkler and Stolzenberg [22] had a higher CPQ score than the children in the middle or high social class. The fundamental cause of this finding could be that caries is increasingly found in socioeconomically compromised families for reasons such as poor oral health education. Thus, the observations in this study overall were similar to preliminary findings [13, 14, 24, 25]. For future studies on this topic, matching participants according to age and sex may not be important, whereas the social status of the participants should still be considered.

Analysis of the tooth-related data revealed that there were clear differences between the caries group and the MIH group. Whereas mostly permanent teeth were affected in the MIH group (86%), deciduous teeth were most affected in the caries group (89%). This discrepancy could be explained by the age of the probands. The development of caries is a progredient process that mainly depends on diet and oral hygiene and only becomes apparent after some time. Since the deciduous molars in this age group are exposed to those contributing factors much longer, the probability of developing caries is higher than in those with recently erupted permanent teeth. In contrast, MIH is, by definition, the most common in the permanent first molars and permanent central incisors [26, 27]. This fact explains why mostly permanent teeth were affected in this group.

Consequently, affected deciduous (caries group) and permanent teeth (MIH group) were compared in terms of their influence on the OHRQoL of the probands. Up to this point, no sufficient data has differentiated pain perception and vulnerability between the two dentitions. Hence, further research comparing deciduous and permanent teeth on this issue is necessary.

Furthermore, the children in the MIH group had three times as many frontal teeth affected as those in the caries group. Again, this finding can be explained by the age of the probands because the change of dentition already had occurred in the anterior region. Due to the relatively short time since the permanent incisors had erupted in the oral cavity by that age, they are rarely affected by caries, even in caries-prone children. Thus, emotional and social compromises in quality of life linked to discolored frontal teeth were more likely in the MIH group than in the caries group, which explains why emotional well-being was significantly worse in the HSC MIH group than in the HSC caries group.

Another important aspect is that the present study is the first to use the German version of the CPQ8-10 created by Jokovic [16]. For both diseases, we noticed an almost linear increase in the total CPQ value as the severity category increased. Between the LSC and HSC, the values tripled for both the caries and MIH groups. These results suggest that the scheme used in this study was sound since these differences cannot be explained by mere probability. The mean values were significantly different between the caries and MIH probands in nearly all subcategories, where MIH probands scores were generally higher than those in the caries group.

In both groups, LSC had only a minor influence on OHRQoL. By definition, carious lesions in this group only extend to the outer third of the dentin, which in most cases does not result in pain or other oral symptoms [13, 28, 29]. The same applies to children with MIH teeth in the same severity category. These mildly affected teeth do not tend to show signs of hypersensitivity or changes in form and, consequently, there are usually no severe oral symptoms [10–12, 17].

MIH probands in this severity category showed significantly higher values in the emotional and social well-being subcategories. This can be explained by affected frontal teeth and the fact that discoloration in facial surfaces can lead to insecurities, especially if they are pointed out by other people.

In the MSC, clear differences between the caries- and MIH-affected children could be observed. The mean total CPQ value was 1.5 times higher for the MIH probands. In particular, increasing severity caused oral health to worsen in the MIH group. Cold-sensitive molars in the higher severity categories, according to Mathu-Muju and Wright, clearly increase “oral symptoms” and “functional limitations” [9, 11, 12, 17–19, 21, 30]. The children in the MIH group had significantly more issues with food intake compared to those in the caries group since caries that reach only the middle third of the dentin (D2) do not cause major symptoms [19]. Additionally, in the MSC category, the MIH children had higher values in the emotional and social subcategories compared to the caries-affected children, which could again be explained by the larger number of affected frontal teeth. The results in this severity category seem to support the conclusion that the medium clinical severity had a greater negative impact on the MIH group than the caries group.

In the HSC, a major increase was noticed in the overall CPQ values in both groups that was double or triple that of the other severity categories. Therefore, a significant increase in values could also be seen in their respective subcategories. Highly affected teeth caused severe oral symptoms and functional limitations. In the oral symptom subcategory, no significant differences between the two groups were noted. The increased number of damaged molars caused more problems with food intake, especially in the MIH group. This finding may be related to the fact that the development of caries is a progredient process to which a child has time to adapt. Strategies can be developed to avoid pain by, for example, changing chewing habits, whereas the sometimes heavily damaged MIH teeth erupt rather quickly and cause immediate pain, resulting in a shorter period of adaptation. Therefore, it was expected that MIH-affected children would have a higher CPQ score than caries-affected children.

For 88% of the MIH and 70% of the caries probands, their overall health status was rated as “excellent” or “very good,” respectively. In contrast, the MIH group members described their oral health as slightly worse than those of the caries group, with 30% of the MIH- and 36% of the caries-affected participants rating it as “excellent” or “very good.” The results show that children that age are indeed able to differentiate between their general and oral health. The large discrepancy between these ratings proves how extensively both diseases influence children’s oral health. It also confirms that for children that age, MIH overall has a more negative impact on OHRQoL than caries.

For various reasons, the results of this study are difficult to compare with those of other studies. One reason is that the German version of the CPQ8-10 had not yet been published. Consequently, no similar data in German-speaking countries could be found. However, even when other countries and cultures were considered, there were few surveys on the effects of caries and MIH on the quality of life of children using the CPQ8-10. These few studies drastically vary in their designs, making a comparison of the results barely possible [9, 13, 17, 18, 28]. On the other hand, none of these existing studies compare the OHRQoL of both diseases simultaneously, which emphasizes the importance of these results.

The validity and relevance of the published classification of severity categories for caries is emphasized by the evidence obtained in this study. For the reasons stated above, this study should be evaluated as a pilot study; therefore, further research should be conducted to evaluate and verify the present results.

## Conclusion

This study shows that both diseases, caries, and MIH have a severe influence on the OHRQoL of children in this age group. The symptoms and discomfort increased significantly with increases in the respective severity category. Moreover, the MIH group showed higher CPQ scores in total as well as in almost all domains. Based on these results, however, it cannot be concluded that MIH is a more severe disease than caries. Undeniably, MIH tends to cause more impairments when severity is equal in this age group; nevertheless, these results are only a small insight into all currently existing cases.

Caries can lead to severe effects on the oral health and general well-being of patients when oral hygiene and dietary habits do not change, whereas MIH tends to not progress to the same extent as caries. For both diseases, it is highly recommended to take appropriate therapeutic measures to ensure these children have a good OHRQoL.

## Appendix 1

**Table 8:** Summary of data on 528 participants with caries (*n* = 264) and MIH (*n* = 264) according to the American Dental Association Caries Classification by Young et al. [19] and the MIH Classification by Mathu-Muju and Wright [21]

12,217 teeth													
% (n)													
		MIH				All affected			Caries				
		50.0 (6,114)							50.0 (6,103)				
		0	I	II	III				0	I	II	III	All affected
Deciduous teeth	49.5 (3,029)	95.7 (2,898)	1.4 (88)	0.7 (41)	0.0 (2)	13.7 (131)	Deciduous teeth	50.4 (3,076)	66.3 (2,040)	17.0 (522)	9.4 (290)	7.3 (224)	89.4 (1,036)
Permanent teeth	50.5 (3,085)	73.2 (2,258)	8.7 (532)	2.7 (167)	2.1 (128)	86.3 (827)	Permanent teeth	49.6 (3,027)	96.0 (2,904)	3.4 (102)	0.6 (18)	0.1 (3)	10.6 (123)
Total	100.0 (6,114)	84.3 (5,156)	10.1 (620)	3.4 (208)	2.1 (130)	15.7 (958)	Total	100.0 (6,103)	81.0 (4,944)	10.2 (624)	5.0 (308)	3.7 (227)	19.0 (1,159)

*MIH* molar incisor hypomineralization

## Appendix 2

**Table 9:** Significant differences between the means of total CPQ scores in the caries and MIH groups by age

				95% CI		*p*-value			
Group	Age (years)	n	Mean CPQ	Lower	Upper	7 vs. 8	8 vs. 9	9 vs. 10	Age
Caries	7	80	9.2	7.1	11.3	.282			.447
	8	64	7.4	5.1	9.8		.300		
	9	62	6.7	4.3	9.1			.542	
	10	58	8.7	6.2	11.1				
MIH	7	80	9.1	7.0	11.2	.140			.244
	8	64	11.4	9.1	13.8		.762		
	9	62	10.7	6.2	11.1			.201	
	10	58	12.2	9.7	14.6				

Considered significant if *p* < 0.05

*CPQ* Child Perceptions Questionnaire, *MIH* molar incisor hypomineralization, *CI* confidence interval

## Appendix 3

**Table 10:** Significant differences between means of total CPQ scores in the caries and MIH groups by gender

Group	Gender	*n*	Mean CPQ	95% CI		*p*-value	
				Lower	Upper	Gender	Gender group
Caries	♀	131	7.7	6.0	9.4	.575	.277
	♂	133	8.4	6.8	10.0		
MIH	♀	131	11.2	9.6	12.9	.323	
	♂	133	10.1	8.5	11.8		

Considered significant if *p* < 0.05

*CPQ* Child Perceptions Questionnaire, *MIH* molar incisor hypomineralization

## Appendix 4

**Table 11:** Significant differences between means of total CPQ scores in the caries and MIH groups by social status

Group	Social status	*n*	Mean CPQ	95% CI		*p*-value			
				Lower	Upper	Low vs. medium	Medium vs. high	Social status	Social status* group
Caries	Low	74	14.1	11.9	16.1	.000		.000	
	Medium	130	6.7	5.1	8.3		.000		
	High	60	3.7	1.4	6.0				
MIH	Low	74	12.2	10.1	14.4	.068		.187	.000
	Medium	130	9.8	8.1	11.4		.915		
	High	60	10.9	8.5	13.2				

Considered significant if *p* < 0.05

*CPQ* Child Perceptions Questionnaire, *MIH* molar incisor hypomineralization, *CI* confidence interval

## References

[1] Jordan AR, Micheelis W (2016) Fünfte Deutsche Mundgesundheitsstudie-(DMS IV). Deutscher Zahnärzte Verlag DÄV 10.1186/1472-6831-14-161 PMC4417261 25547464

[2] Micheelis W (1991) Mundgesundheitszustand Und-Verhalten in Der Bundesrepublik Deutschland: Ergebnisse Des Nationalen IDZ-Survey 1989. Dt. Ärzte-Verlag

[3] Schwendicke F, Dörfer CE, Schlattmann P, Foster Page L, Thomson WM, Paris S (2015). Socioeconomic Inequality and Caries: A Systematic Review and Meta-Analysis. J Dent Res.

[4] van der Tas JT, Kragt L, Elfrink MEC (2017). Social inequalities and dental caries in six-year-old children from the Netherlands. J Dent.

[5] Koch G, Hallonsten A-L, Ludvigsson N, Hansson BO, Hoist A, Ullbro C (1987). Epidemiologic study of idiopathic enamel hypomineralization in permanent teeth of Swedish children. Community Dent Oral Epidemiol.

[6] Weerheijm KL, Duggal M, Mejàre I (2003). Judgement criteria for Molar Incisor Hypomincralisation (MIH) in epidemiologic studies: a summary of the European meeting on MIH held in Athens, 2003. Eur J of Paediatr Dent.

[7] Weerheijm KL, Jälevik B, Alaluusua S (2001). Molar–incisor hypomineralisation. Caries Res.

[8] BaniHani A, Deery C, Toumba J, Munyombwe T, Duggal M (2018). The impact of dental caries and its treatment by conventional or biological approaches on the oral health-related quality of life of children and carers. Int J Paediatr Dent.

[9] Portella PD, Menoncin BLV, de Souza JF, de Menezes JVNB, Fraiz FC, Assunção LR d S (2019). Impact of molar incisor hypomineralization on quality of life in children with early mixed dentition: A hierarchical approach. Int J Paediatr Dent..

[10] Dantas-Neta NB, de Moura LFA, Cruz PF, Moura MS, Paiva SM, Martins CC (2016) Impact of molar-incisor hypomineralization on oral health-related quality of life in schoolchildren. Braz Oral Res 30 10.1590/1807-3107BOR-2016.vol30.0117 27783769

[11] Fütterer J, Ebel M, Bekes K, Klode C, Hirsch C (2020). Influence of customized therapy for molar incisor hypomineralization on children’s oral hygiene and quality of life. Clin and Exp Dent Res.

[12] Ebel M, Bekes K, Klode C, Hirsch C (2018). The severity and degree of hypomineralisation in teeth and its influence on oral hygiene and caries prevalence in children. Int J Paediatr Dent..

[13] Mota-Veloso I, Soares MEC, Alencar BM, Marques LS, Ramos-Jorge ML, Ramos-Jorge J (2016). Impact of untreated dental caries and its clinical consequences on the oral health-related quality of life of schoolchildren aged 8–10 years. Qual Life Res.

[14] Krisdapong S, Prasertsom P, Rattanarangsima K, Sheiham A (2013). School absence due to toothache associated with sociodemographic factors, dental caries status, and oral health-related quality of life in 12- and 15-year-old Thai children. J Public Health Dent.

[15] Jokovic A, Locker D, Stephens M, Kenny D, Tompson B, Guyatt G (2002). Validity and reliability of a questionnaire for measuring child oral-health-related quality of life. J Dent Res.

[16] Jokovic A, Locker D, Tompson B, Guyatt G (2004). Questionnaire for measuring oral health-related quality of life in eight-to ten-year-old children. Pediatr Dent.

[17] Velandia LM, Álvarez LV, Mejía LP, Rodríguez MJ (2018). Oral health-related quality of life in Colombian children with Molar-Incisor Hypomineralization. Acta Odontol Latinoam.

[18] Gutiérrez TV, Ortega CCB, Pérez NP, Pérez AG (2019). Impact of Molar Incisor Hypomineralization on Oral Health-Related Quality of Life in Mexican Schoolchildren. J Clin Pediatr Dent.

[19] Young DA, Nový BB, Zeller GG, Hale R, Hart TC, Truelove EL, Ekstrand KR, Featherstone JDB, Fontana M, Ismail A, Kuehne J, Longbottom C, Pitts N, Sarrett DC, Wright T, Mark AM, Beltran-Aguilar E (2015). The American Dental Association caries classification system for clinical practice: a report of the American Dental Association Council on Scientific Affairs. J Am Dent Assoc.

[20] Campus G, Cocco F, Ottolenghi L, Cagetti MG (2019) Comparison of ICDAS, CAST, Nyvad’s Criteria, and WHO-DMFT for Caries Detection in a Sample of Italian Schoolchildren. Int J Environ Res and Public Health. 16 10.3390/ijerph16214120 PMC6862073 31731559

[21] Mathu-Muju K, Wright JT (2006). Diagnosis and treatment of molar incisor hypomineralization. Compend Contin Educ Dent.

[22] Winkler J, Stolzenberg H (2009) Adjustierung Des Sozialen-Schicht-Index Für Die Anwendung Im Kinder-Und Jugendgesundheitssurvey (KiGGS). Wismarer Diskussionspapiere

[23] Bekes K, Ebel M, Omara M et al (2020) The German version of Child Perceptions Questionnaire for children aged 8 to 10 years (CPQ-G8-10): Translation, reliability, and validity. Clin Oral Inv:1–7 10.1007/s00784-020-03451-w PMC7878203 32666348

[24] Ghasemianpour M, Bakhshandeh S, Shirvani A, Emadi N, Samadzadeh H, Moosavi Fatemi N, Ghasemian A (2019). Dental caries experience and socio-economic status among Iranian children: a multilevel analysis. BMC Public Health.

[25] de la Cruz P, Silvia CJ (2020). Oral Health Problems and Utilization of Dental Services Among Spanish and Immigrant Children and Adolescents. Int J of Environ Res Public Health.

[26] Ghanim A, Mariño R, Manton DJ (2019). Validity and reproducibility testing of the Molar Incisor Hypomineralisation (MIH) Index. Int J Paediatr Dent.

[27] Mittal N, Sharma BB (2015). Hypomineralised second primary molars: prevalence, defect characteristics and possible association with Molar Incisor Hypomineralisation in Indian children. Eur Arch Paediatr Dent.

[28] de Souza Barbosa T, de Morais Tureli MC, Nobre-dos-Santos M, Puppin-Rontani RM, Gavião MBD (2013). The relationship between oral conditions, masticatory performance and oral health-related quality of life in children. Arch Oral Biol.

[29] Shin H-S, Han D-H, Shin M-S, Lee H-J, Kim M-S, Kim H-D (2015). Korean version of child perceptions questionnaire and dental caries among Korean children. PloS One..

[30] Wright JT (2015) Diagnosis and treatment of molar-incisor hypomineralization. Handbook of Clinical Techniques in Pediatric Dentistry. Wiley Blackwell, pp 99–106

